# Complex Ocular Injury Following Blunt Trauma in a Professional Adult Male Soccer Player: A Case Report

**DOI:** 10.7759/cureus.71677

**Published:** 2024-10-17

**Authors:** Alexis Lauga, Adam Maguire, Mahi Muqit

**Affiliations:** 1 School of Clinical Medicine, University of Cambridge, Cambridge, GBR; 2 Academy Medical Department, Tottenham Hotspur Football Club, London, GBR; 3 Vitreoretinal Service, Moorfields Eye Hospital, London, GBR; 4 Institute of Ophthalmology, University College London, London, GBR

**Keywords:** blunt ocular trauma, eye injury, ophthalmology, soccer, sports medicine

## Abstract

This case describes an 18-year-old British male professional soccer player who suffered a high-velocity traumatic eye injury when a football struck him in the right eye. He suffered immediate vision loss and pain. The eye trauma resulted in pupil damage, lens structural damage, retinal tears, a macular hole, and extensive retina commotio. Despite being a seemingly innocuous training accident, he developed a complex monocular trauma. He underwent emergency surgery that successfully closed the macular hole and prevented retinal detachment. Visual recovery in the right eye was not perfect; however, the player was able to make a full return to training within six weeks and play competitive football within eight weeks. This exemplifies a rare case in which a routine, everyday training ground event resulted in a highly complex ocular injury. Our case highlights that rapid assessment and intervention can prevent life-changing long-term effects on an athlete’s health, performance, and career, even in sports deemed to pose a relatively low risk of ocular injury, such as soccer.

## Introduction

The World Health Organization (WHO) estimates 55 million eye injuries presenting to emergency departments (EDs) globally every year, 750,000 of which require hospitalisation [1]. Sports account for up to a quarter of these cases, with ocular trauma being the second most prevalent form of injury in sports after musculoskeletal injury [2].

Although soccer is deemed to have only a ‘moderate risk’ of ocular injury relative to other sports, across Europe, it is the single most common cause of sports-related eye injuries [3,4]. One study found it to account for 21% of complex ocular trauma cases with severe consequences, the most of any individual sport [5]. In soccer, roughly 80% of eye injuries occur in men under 25 and are monocular injuries that result from blunt trauma, most commonly secondary to a high-velocity trauma from a kicked ball [4,6].

In this paper, we present the case of an 18-year-old male who incurred blunt trauma to the right eye while playing a soccer match. We discuss the severe and complex ocular injury that ensued, focusing on the signs and symptoms, diagnosis, and management of each injured sub-structure of the eye. We report a rare combination of injuries and an even rarer case of successful surgical intervention following such severe trauma, which resulted in a quick return to elite sport. Finally, we discuss the existing guidelines on return to play following an eye injury in professional soccer leagues.

## Case presentation

**Figure 1:**
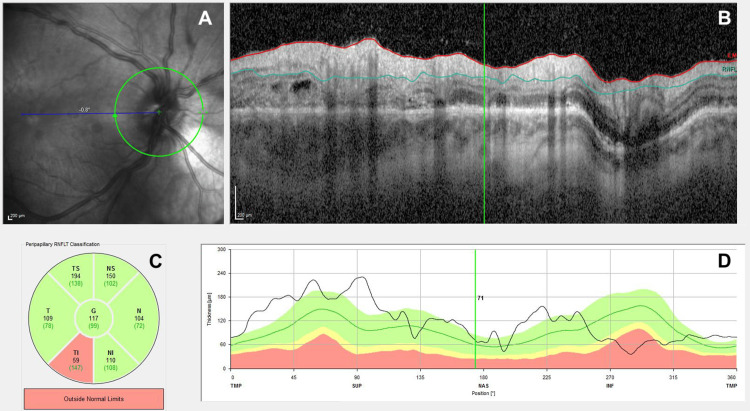
Circular peripapillary OCT images showing macula commotio in the right eye. Images taken on the 30th of August 2022, three days before surgery. There is obvious commotio affecting the macula across its diameter. (A) Image obtained using dual-beam scanning laser ophthalmoscopy. (B) Corresponding fundus image. (C) Mean RNFLT plotted in the main areas of the eye. (D) RNFLT plotted on the thickness values measured in healthy subjects of the same age. OCT: optical coherence tomography; RNFLT: retinal nerve fibre layer thickness; NAS: nasal; SUP: superior; INF: inferior; TMP: temporal; ILM: internal limiting membrane; T: temporal; N: nasal; TS: temporal-superior; TI: temporal-inferior; NS: nasal-superior; NI: nasal-inferior; G: global value

An 18-year-old British male professional soccer player suffered a severe traumatic eye injury during a match when the ball ricocheted off an opponent’s foot and struck him in the right eye at high velocity. He immediately complained of poor visual acuity and pain unilaterally. On examination by the team doctor, he was only able to count fingers with the right eye and there was evidence of periocular swelling and traumatic mydriasis with a sluggish pupillary response. The left eye was healthy with 6/6 vision. He was given paracetamol and seen in the local eye casualty by an on-call ophthalmologist, who treated him with topical therapy. Four days later, he was evaluated by a retinal surgeon. Post-injury, the visual acuity had worsened to the level of hand motions. A slit-lamp examination revealed that the vitreous gel prolapsed into the upper section of the anterior chamber through a zonular defect, accompanied by low eye pressure at 6 mmHg. Together, this suggested trauma to the crystalline lens zonular apparatus and some ciliary body shock with reduced aqueous production. The lens itself was not mobile. Detailed ultrasound examination revealed incomplete vitreoschisis, incomplete posterior vitreous detachment (PVD) from the macula and retina with impending collapse of the vitreous anteriorly, as well as moderate vitreous haemorrhage. Scleral indentation retinal examination identified a retinal tear with overlying traction in the superiotemporal quadrant. There was widespread retina and macula commotio, seen as whitening and opacification of the neuroretina (Figure 1).

**Figure 2:**
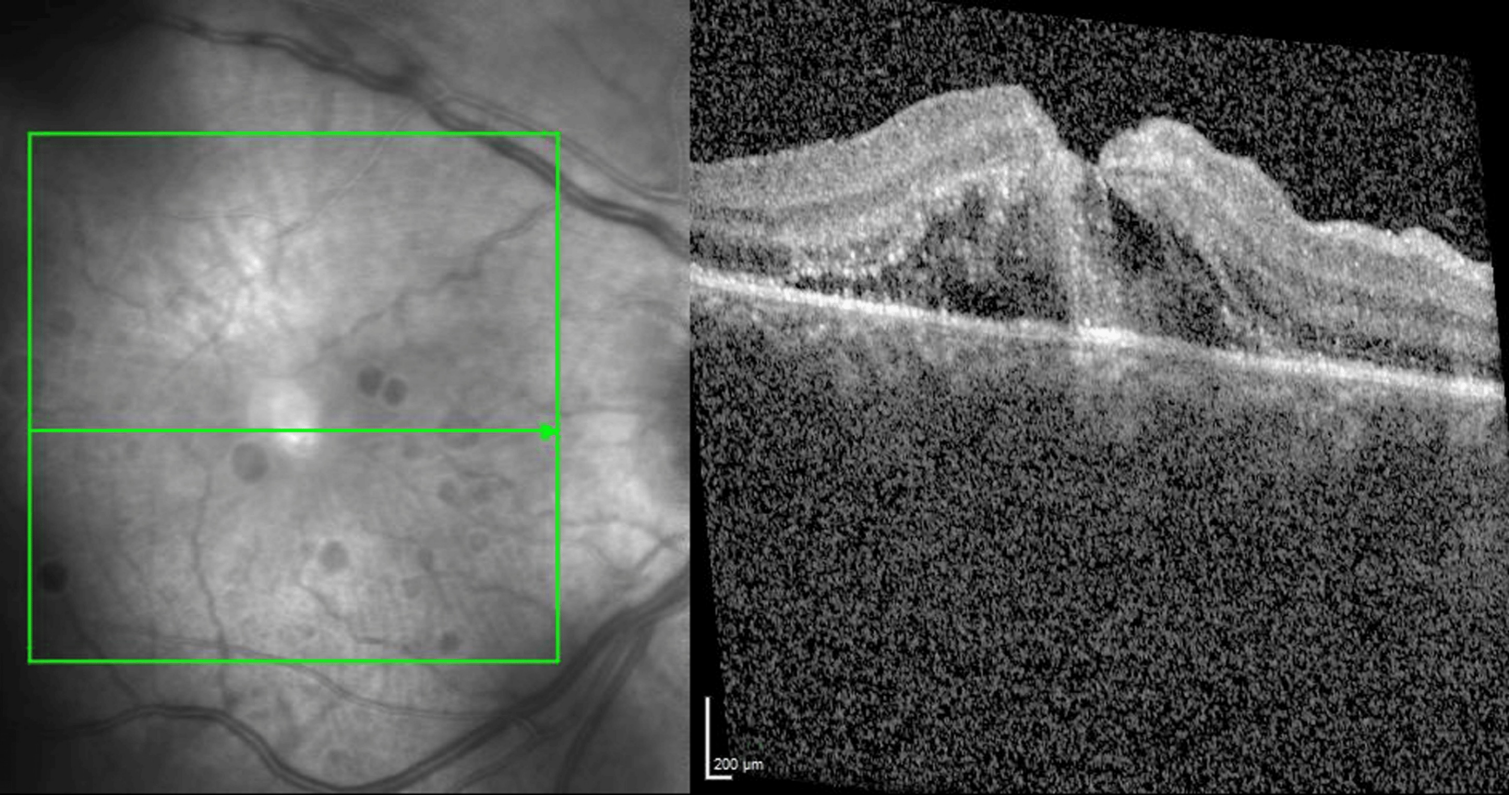
OCT images showing central macular oedema with an impending macular hole in the right eye. Images taken on the 30th of August 2022, three days before surgery. There is evidence of disruption and thinning of the central fovea with an impending macular hole. OCT: optical coherence tomography

The optical coherence tomography (OCT) imaging revealed central macular oedema, with disruption and thinning of the central fovea (Figure 2). The optic nerve did not show any signs of traumatic avulsion. However, there was peripapillary swelling and commotio, as well as a degree of nerve fibre layer damage in one sector. A mild afferent pupillary defect was present. His traumatic macular condition progressed from oedema and foveal thinning to an impending full-thickness macular hole. This explained his decline in central visual acuity, which is the hallmark symptom of this condition. He was diagnosed with a complex and severe monocular trauma, necessitating surgical treatment. Without surgery for the macular condition, he would likely have developed a macular hole and permanent scarring of the central macula, resulting in a poor visual outcome. The surgeon discussed all the different possible options with the patient and team doctor. Given the evolving central foveal complications and impending macular hole, combined with the vitreous haemorrhage and tractional retinal break, it was decided that the player would undergo emergency vitreoretinal surgery to correct all abnormalities.

**Figure 3:**
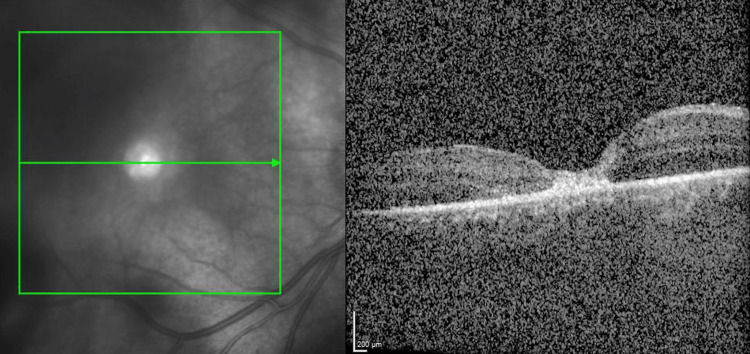
OCT images showing close apposition of the outer retinal layers following surgery. Images taken on the 2nd of September 2022, postoperatively. OCT: optical coherence tomography

His peripheral retinal break underwent cryosurgical repair, accompanied by a gas-fluid exchange with short-acting 20% sulfahexafluoride gas tamponade to prevent retinal detachment and treat the impending macular hole. The internal limiting membrane was peeled at the central macula, and the operation was uncomplicated. Throughout the course of the gas bubble’s disappearance over the next three weeks, the retina remained attached in all quadrants. The surgery allowed for close apposition of the outer retinal layers (Figure 3). Within two weeks, the player’s macular hole had sealed completely.

**Figure 4:**
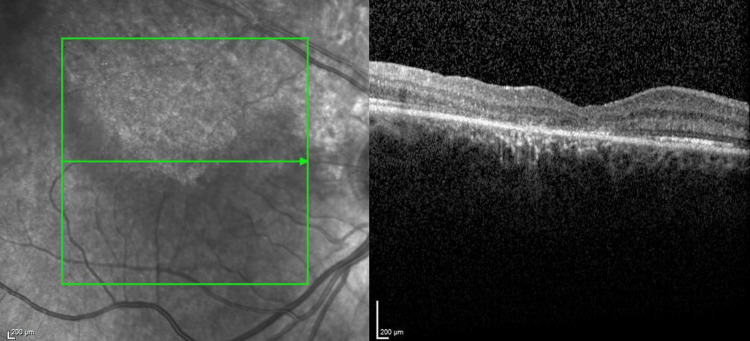
OCT images showing healing of the retina across its thickness. Images taken on the 22nd of May 2023, nearly nine months after surgery. OCT: optical coherence tomography

Regular OCT monitoring subsequently showed gradual inner and outer retinal healing (Figure 4). The central vision in his right eye progressively improved over the course of the next six months to 20/100.

**Figure 5:**
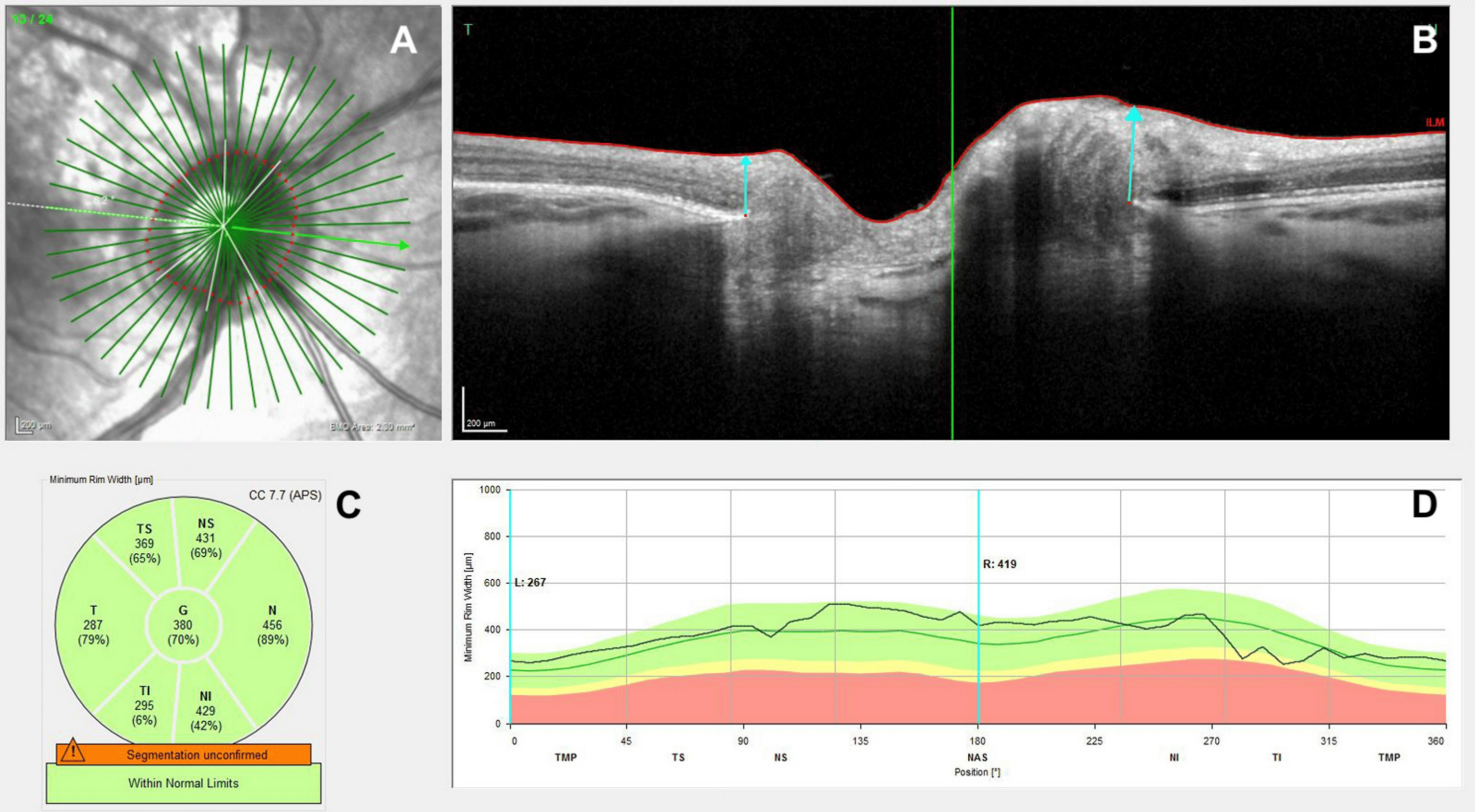
OCT images showing the resolution of macula commotio in the right eye. Images taken on the 23rd of May 2024, nearly 21 months after surgery. (A) Image obtained using dual-beam scanning laser ophthalmoscopy. (B) Corresponding fundus image. (C) Mean RNFLT plotted in the main areas of the eye. (D) RNFLT plotted on the thickness values measured in healthy subjects of the same age. OCT: optical coherence tomography; RNFLT: retinal nerve fibre layer thickness; NAS: nasal; SUP: superior; INF: inferior; TMP: temporal; ILM: internal limiting membrane; T: temporal; N: nasal; TS: temporal-superior; TI: temporal-inferior; NS: nasal-superior; NI: nasal-inferior; G: global value; CC: centrocecal; APS: anatomic positioning system

The right eye pressure had returned to normal within two weeks. One month later, the intraocular pressure (IOP) was found to be elevated at 22 mmHg. This was corrected using timolol, and a glaucoma specialist confirmed there to be no damage to the optic disc. The IOPs equalised around six weeks after surgery and remained normal thereafter. The lens maintained stability throughout, and the timolol was weaned down and stopped. At eight months following surgery, his eye pressure remained normal, and the optic nerve structure had been fully restored, with a normal optic nerve fibre layer scan (Figure 5).

## Discussion

In soccer, it is not uncommon for the ball to be kicked into a player’s face and eye. Though this is usually an innocuous trauma, there exist patients who present with complex, severe ocular injury, as illustrated by the player discussed. Given the diagnosis of an impending macular hole, traumatic commotio, retinal tear, vitreous haemorrhage, and damage to the crystalline lens zonule, surgery is essential to rescue ocular function and reverse traumatic structural changes. The long-term visual prognosis depends on how much the retina recovers and how the anterior ocular structures behave over the following months.

Macular oedema and impending traumatic macular hole

A macular hole is a full-thickness breach of the neuroretina that disrupts the foveal contour. Although usually idiopathic or age-related, it may occur as a complication of ocular trauma and is found in 1.4% of cases of blunt, closed-globe injuries [7].

Those with a macular hole conventionally undergo vitrectomy with a macular peel. This is followed by the replacement of the vitreous with a gas bubble. Its surface tension allows for close apposition of the torn edges whilst the retina heals. This process takes up to 10 weeks, with 75% of traumatic macular hole cases closing within three months of surgery and a further 8% closing within a year [8]. In some patients, watchful waiting is preferred to surgery as the macular hole may close spontaneously over the next six months. However, this conservative approach only shows a 40% rate of closure, with the majority occurring in the first three months [9]. The traumatic macular holes that do not close spontaneously result in poor visual outcomes. Therefore, guidelines may choose to advise different strategies according to injury severity. In patients with an isolated macular hole, surgery might be delayed for up to three months whilst the retina is observed for spontaneous healing, after which surgery should be considered. In more complex cases of trauma, emergency surgery should instead be offered first-line, as was the case for this patient.

Retinal breaks

Up to 40% of retinal tears occur secondary to trauma, most of which involve blunt, closed-globe injuries [10]. Roughly 10% of sports-related ocular traumas involve a retinal tear which is one of the most prevalent eye injuries in soccer [4]. Compression of the globe increases the IOP and places the retina and its blood vessels under vitreous traction, which causes a tear with accompanying vitreous haemorrhage. Soccer players and other endurance athletes show sustained increases in their IOPs and are therefore at an increased risk of retinal breaks [11].

Patients with an acute retinal tear accompanied by vitreous haemorrhage are rarely asymptomatic. They often complain of myodesopsias (floaters), photopsias (flashes), or reduced visual acuity [12]. Such symptomatic cases carry the highest risk of progression to retinal detachment and are therefore treated surgically. The choice of treatment is dictated by the cause and location of the lesion. Laser photocoagulation is usually reserved for central tears caused by proliferative vasculopathies, while cryopexy is more suited to peripheral retinal breaks resulting from trauma.

Retina and macula commotio

Commotio is a common finding in closed-globe injuries and is estimated to be present in up to 42% of cases of blunt trauma involving sports [6]. One study found it to be the single most common injury among soccer-related ocular traumas [13]. Histopathological analysis has shown this to result from photoreceptor reorientation and subsequent scattering of light, leading to a whitening effect [14]. Retina commotio is typically asymptomatic as it usually involves the peripheral retina, although some patients have a slightly restricted visual field [6]. Treatment for peripheral commotio is generally expectant as the risk of sequelae is low, with most patients regaining at least some visual acuity within four weeks [15]. In cases where commotio is associated with traumatic iritis, additional use of topical corticosteroids is recommended [6]. Most patients report a final visual acuity better than 20/30 [16]. Cases involving the macula are more commonly symptomatic and patients complain of transient changes such as blurry central vision or metamorphopsia, while others describe scotomas [15]. A portion of such patients report persistent blurriness, scotomas, or metamorphopsia, despite apparent resolution of commotio on OCT.

Trauma to the crystalline lens zonule

Zonular injury is present in up to two-thirds of closed-globe injuries but is missed in up to 57% of clinical examinations, with the remainder only being diagnosed at the time of surgery [17]. This can be attributed to factors such as opacification of the anterior chamber (such as hyphaema) and injuries that do not reach the zonule damage threshold for lens subluxation, as demonstrated by this patient. A missed/late diagnosis may lead to complications during surgical treatment acutely after the trauma, as well as in later procedures such as cataract surgery. Ultrasound biomicroscopy (UBM) is highly effective in assessing zonular damage before surgery. In cases of complex trauma, UBM may guide the usage of gas tamponades, which further increase the risk of phacodonesis.

Such cases of mild zonulopathy, where there is no subluxation or dislocation of the lens, are commonly asymptomatic, with only a few recorded cases of decreased visual acuity. Treatment is generally conservative, with the lens being monitored regularly for phacodonesis. Any dysregulation in IOP is treated accordingly by the use of topical agents.

Player recovery

Despite the severity of the injury, the player made an exceptionally quick recovery with light activity allowed after six weeks and a full return to play with contact and heading after just eight weeks. Twenty months down the line, the player is still under review. He was able to make a full return to play with protective eyewear. Following a discussion on the risks of playing without, the player decided to stop using the eyewear after a few weeks. While his vision is by no means perfect in his right eye (20/100), it does not cause him too much bother and he is able to drive. He has been noted to have a small degree of subcapsular lens opacity, indicating a mild cataract. It was chosen not to correct this surgically as it does not affect his ability to see the ball and the lens is currently stable. Were he to suffer from traumatic lens dislocation into the vitreous cavity down the line, there are several surgical solutions that can be undertaken to restore vision and insert a special lens implant.

Guidelines on return to play

There are currently no published guidelines outlining when it is safe for a soccer player to return following an eye injury. The decision is instead left to the discretion of the team doctor(s) and the ophthalmologist, in conjunction with the player’s wishes. Patient clearance should account for both the risk of re-injury and the player’s ability to play at a sufficiently high level without pain or discomfort.

Very few professional sports leagues globally mandate the use of protective eyewear, with even fewer standards at the semi-professional and amateur levels. The latest policy in the United States recommends that protection be worn in players who wear prescription lenses (which should be polycarbonate), who have had a refractive surgical procedure that weakens the eye, or who are functionally one-eyed (where the corrected visual acuity is 20/40 or worse in the affected eye) [3]. Additionally, eyewear should conform to the standards set by the American Society for Testing and Materials (ASTM). In soccer, it is recommended that ASTM F803 be worn as part of a close-fitting frame that does not affect the projection of the ball.

In the United Kingdom’s professional soccer leagues, including the Premier League, there is no clear pathway to assess a player’s fitness to play following a head injury that includes ocular trauma. Neuropsychological testing is performed pre-season and acts as a baseline in the event of injury. However, this does not include formal visual testing, and in the event of an ocular injury, the decision of when a player should be allowed to return to play remains at the discretion of the team doctor in discussion with the ophthalmological specialist.

## Conclusions

Complex ocular trauma should never be ruled out based on the perceived innocuous nature of an eye injury. This case highlights the importance of thorough investigation and management of seemingly innocuous ocular traumas in sports. The patient in this case suffered a severe injury but was able to continue his career thanks to prompt assessment and timely intervention. Guidelines on return to play and wearing of protective eyewear following ocular trauma should be reviewed.
